# Supplementary material for: Nb-Based Catalysts for the Valorization of Furfural into Valuable Product through in One-Pot Reaction
Source: Int J Mol Sci. 2024 Feb 23;25(5):2620. doi: 10.3390/ijms25052620 (PMC10931813; doi:10.3390/ijms25052620)
Supplement: Supplementary file 1 [file ijms-25-02620-s001.zip › ijms-2853110-supplementary.pdf]

Supplementary Information

Small-angle X-ray scattering (SAXS) measurements were performed on a D8 DISCOVER-Bruker instrument at 40 kV and 40 mA. Powder patterns were recorded in capillary transmission configuration by using a Göbel mirror (Cu Kα1 radiation) and the LYNXeye detector. The powder patterns were recorded between 0.2 and 10 º in 2θ with a total measuring time of 120 min.

Powder X-ray diffraction was used to study the crystallinity of Nb-based catalysts supported on porous silicas, with a PANalytical X'Pert PRO diffractometer, with a germanium monochromator and Cu Kα (1.5406 Å) radiation.

The catalyst morphology was evaluated by TEM-EDS, with a FEI Talos F200X, which combines a high-resolution STEM, TEM imaging, and an energy dispersive X-ray spectroscopy (EDS) signal detection. The 3D chemical characterization was obtained from the compositional mapping. The samples were dispersed in ethanol and a drop of the suspension was put on a Formvar/carbon supported Cu grid (300 mesh).

Textural parameters were analyzed by adsorption-desorption of N_2 at -196 °C, using an automatic ASAP 2020 Micromeritics. Samples were previously outgassed overnight at 150 °C and 10^{-4} mbar. The de Boer's t-plot method was used to obtain the micropore surface areas, whereas the specific surface area was deduced from the Brunauer-Emmett-Teller equation (BET), considering a N_2 cross section of 16.2 Å^2. The Nonlocal Density Functional Theory (NLDFT) was applied to determine the pore size distribution from the desorption branch of the isotherm. The total pore volume was deduced from N_2 adsorbed at P/P_0 = 0.996.

A Physical Electronics PHI5700 spectrometer, with non-monochromatic Mg Kα radiation (300 W, 15 kV, and 1253.6 eV) and a multichannel detector, was employed to obtain the X-ray photoelectron spectra. A constant pass energy mode at 29.35 eV, with a 720 μm diameter analysis area, was used for recording the spectra. Acquisition and data analysis were performed with a PHI ACCESS ESCA-V6.0F software package, whereas charge referencing was measured against adventitious carbon (C 1s at 284.8 eV). A Shirley-type background was subtracted from the signals, and the fitting of recorded spectra was carried out with Gaussian-Lorentzian curves, for a better determination of different binding energies.

The total amount of acid sites was determined by thermo-programmed desorption (NH_3-TPD) in a AutoChem II 2920 equipment. For each study, about 50 mg of sample was cleaned with He flow (50 mL/min) with a rate of 30 ºC/min until 550 ºC, maintaining this temperature for 15 min. Then, the sample was cooled down to 80 ºC in He under the same flow. In the next step, the sample was undergone to a NH_3/He (10% vol. NH_3) flow (50 mL/min) at 80 ºC for 20 min. After that, a flow of He of was passed 50 mL/min to 90 min, to eliminate the physisorbed NH_3. Finally, the sample was heated from 80 to 550 ºC, monitoring the NH_3-desorpbed by thermal conductivity detector (TCD).

FTIR spectra of adsorbed pyridine were recorded on aVertex70 FT-IR spectrophotometer model supplied by Bruker. Self-supported wafers of the samples with a weight/surface radio of approximately 15 mg/cm^2 were placed in a vacuum cell with greaseless stopcocks and CaF_2 windows. The samples were evacuated at 250 ºC and 10^{-2} Pa overnight, before they were exposed to pyridine vapors at room temperature for 15 min and then outgassed at 200 ºC.

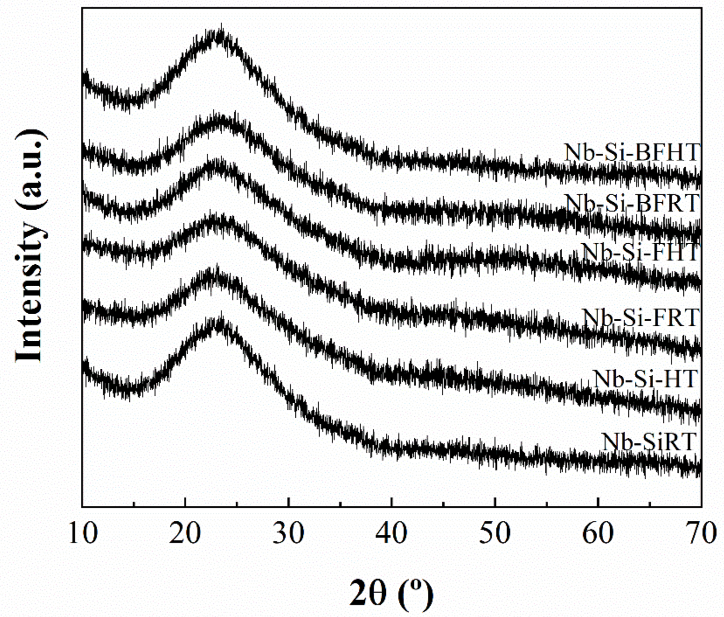

Figure S1. X-ray diffractograms of Nb-based catalysts supported on porous silicas.

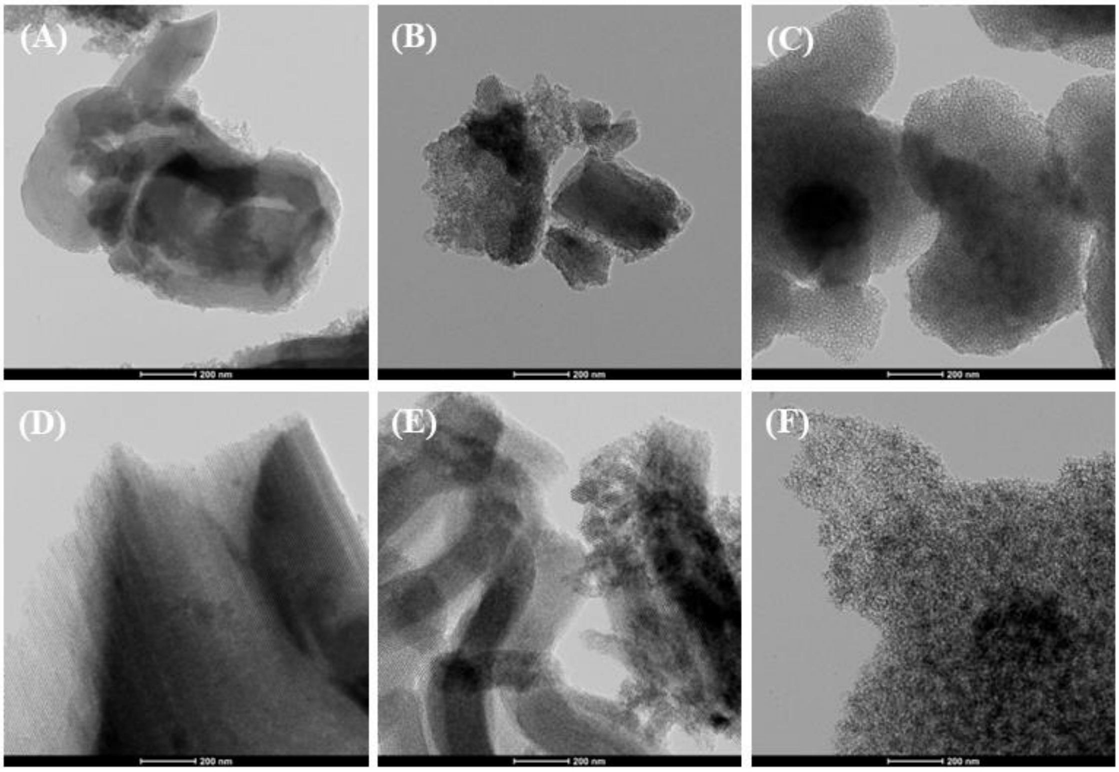

Figure S2. TEM micrographs of the Nb-based catalysts supported on porous silicas (Scale: 200 nm). A) Nb-Si-RT, B) Nb-Si-FRT, C) Nb-Si-BFRT, D) Nb-Si-HT, E) Nb-Si-FHT and F) Nb-Si-BFHT.

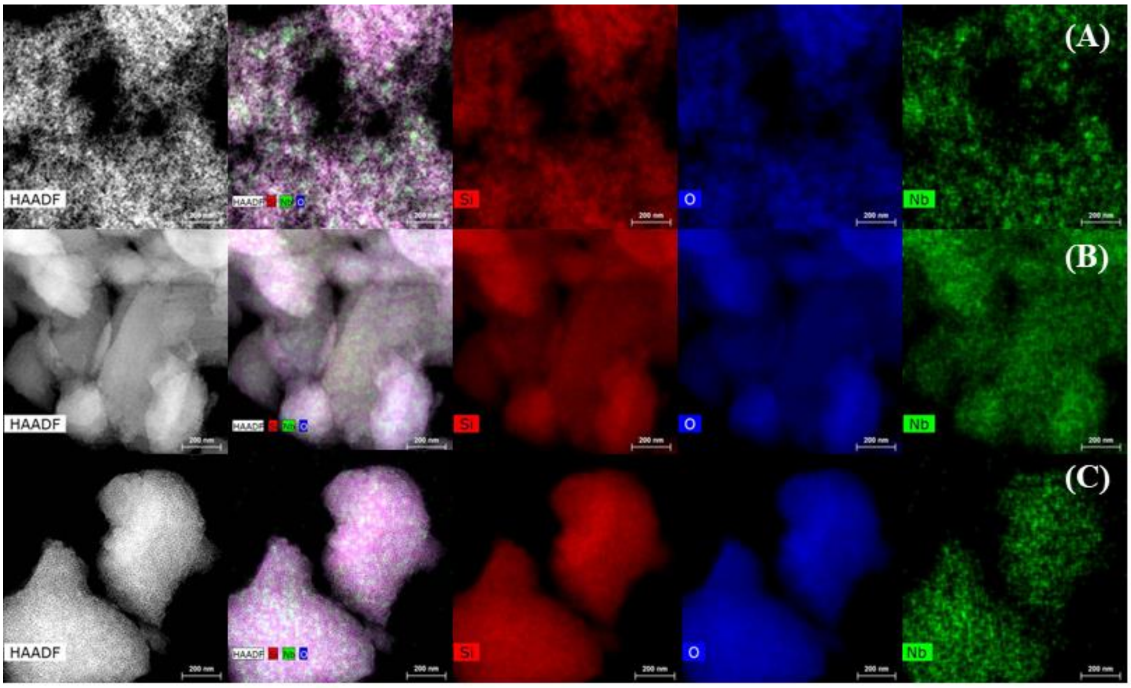

Figure S3. Mapping obtained from S-TEM micrographs of A) Nb-Si-RT, B) Nb-Si-FRT, and C) Nb-Si-BFRT catalysts. (Scale: 200 nm).

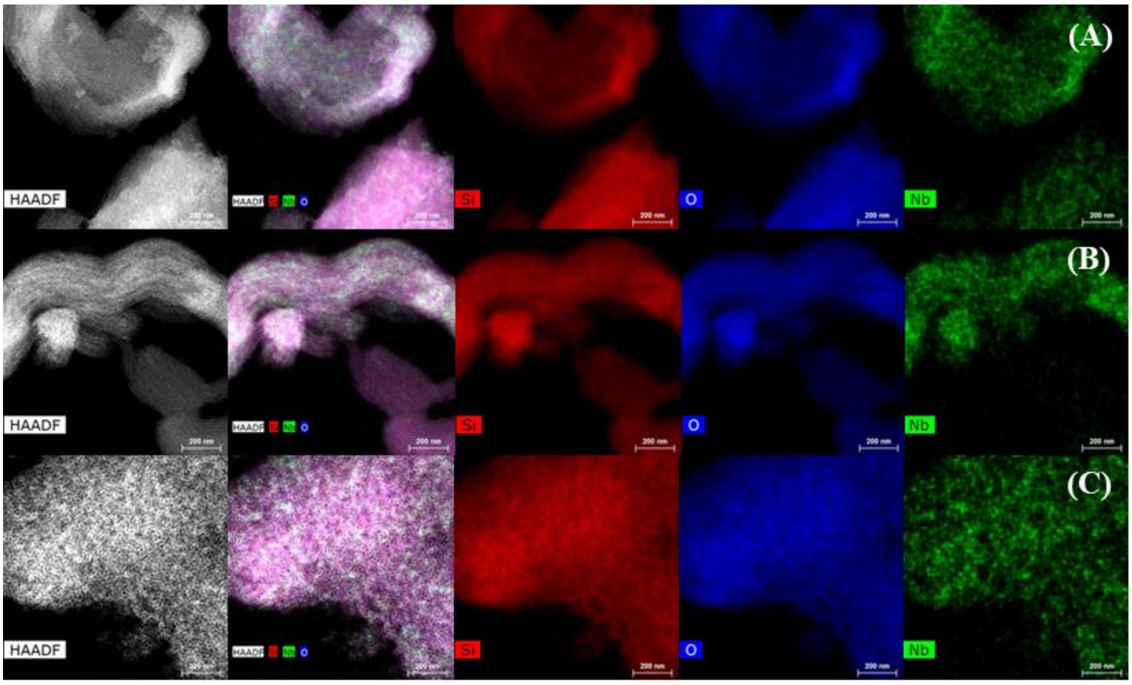

Figure S4. Mapping obtained from S-TEM micrographs of A) Nb-Si-HT, B) Nb-Si-FHT, and C) Nb-Si-BFHT catalysts. (Scale: 200 nm).

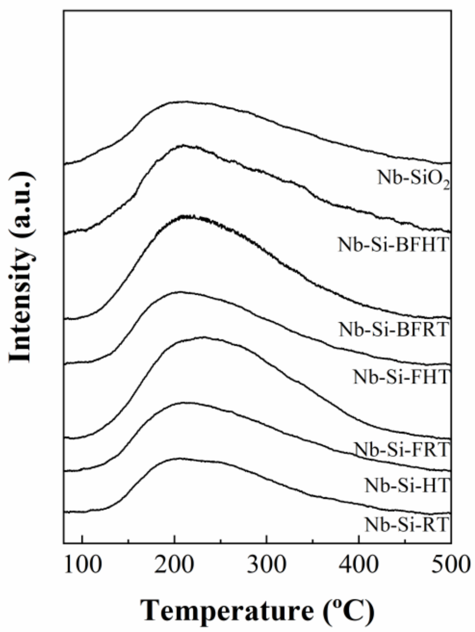

Figure S5. NH_3-Thermoprogrammed desorption of Nb-based catalysts supported on porous silicas.

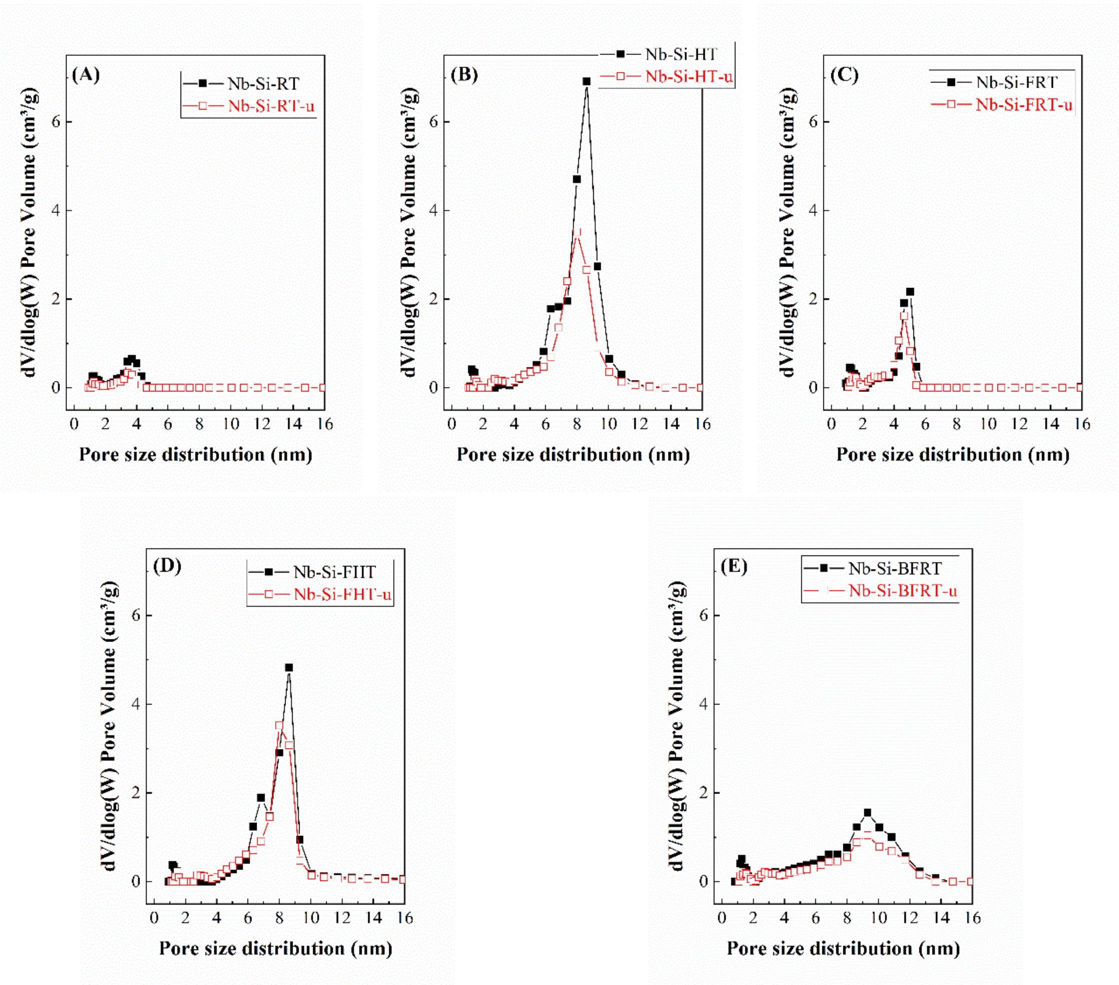

Figure S6. Comparison of the pore size distribution estimated by the DFT method for the Nb-based catalysts supported on porous silica before and after the reaction at 170 ºC for (A) Nb-Si-RT, (B) Nb-Si-HT, (C) Nb-Si-FRT, (D) Nb-Si-FHT and (E) Nb-Si-BFRT catalysts.
